# A comprehensive overview of patient journey and management decision pathway for myelofibrosis in India: INLAND survey

**DOI:** 10.1186/s12885-025-14476-3

**Published:** 2025-07-10

**Authors:** Prantar Chakrabarti, Abhay Bhave, Claire Harrison, Tulika Seth, Vikram Mathews, Disha Shetty

**Affiliations:** 1 Consultant Haematologist, Zoho Corporation, Kolkata, India; 2 Empire Centre Haematology & Onocology Speciality Clinic, Mumbai, India; 3 Data and Analytics, Guy’s and St Thomas’ NHS Foundation Trust, London, UK; 4 AIIMS, New Delhi, India; 5 Christian Medical College, Vellore, India; 6 Novartis healthcare Private Limited India, Mumbai, India

**Keywords:** Myelofibrosis, Myeloproliferative neoplasms, Symptom burden, Treatment goal

## Abstract

**Background:**

Myelofibrosis is a myeloproliferative neoplasm characterized by stem cell-derived clonal myeloproliferative and anomalous production of cytokines with genetic mutations in the JAK/STAT signalling pathway playing a distinctive role in its pathophysiology. Diagnosis of MF presents a challenge due to vague and overlapping symptoms. The present strategy for managing MF is not well defined and relies on a symptomatic approach. Ruxolitinib is the first drug approved by the United States Food and Drug Administration in 2011. Drug Controller General of India approved Ruxolitinib in 2013 as a first-line treatment in patients with MF. This comprehensive survey aims to understand the MF patient journey and the perceptions/practices of Indian hematologists/oncologists regarding diagnosis, prognosis, and disease management of myelofibrosis.

**Methods:**

A cross-sectional, multicentric, qualitative survey was conducted across 17 Indian cities from October 2021 to November 2021. One-on-one telephonic interviews were conducted using a structured questionnaire based on the study objective. Descriptive statistics were used to analyse the obtained data.

**Results:**

Overall, 50 physicians and 154 patients (primary MF: 51, post-PV: 78 and post-ET: 25) completed the survey. The most common symptoms reported by patients and physicians at the time of diagnosis were, abdominal pain/discomfort (81% vs. 70%), fatigue/tiredness/weakness (77% vs. 73%) and fever (54% vs. 48%). A 10-month delay was observed from the symptom onset to the final diagnosis and a further 9-month lag from final diagnosis to treatment initiation. In our survey, the physicians preferred Hydroxyurea (88%), blood transfusion (82%) and Ruxolitinib (78%) as the treatment regimen. The majority of the patients were aware of their treatment. Hydroxyurea constituted the predominant treatment option (*n* = 85); however, satisfaction was highest with Ruxolitinib (50%, *n* = 13). The physicians considered improving overall survival as an important treatment goal, while patients prioritized symptom relief.

**Conclusion:**

The survey highlights the importance of understanding symptom burden and treatment goal perceptions in shaping management decisions. The results emphasize the need to align the physicians and patients on the actual treatment outcomes through patient education. Strengthening this alignment will enhance treatment adherence, improve patient satisfaction, and ensure better clinical outcomes, ultimately leading to a more patient-centred approach to managing the disease.

**Supplementary Information:**

The online version contains supplementary material available at 10.1186/s12885-025-14476-3.

## Background

Myelofibrosis (MF) is a type of myeloproliferative neoplasm (MPN) characterized by stem cell-derived clonal myeloproliferation and anomalous production of cytokines [1, 2]. According to the World Health Organization classification of MPNs, the primary MF, polycythemia vera (PV), and essential thrombocythemia (ET) are grouped as the Janus kinase 2 (JAK2) MPNs, with genetic mutations playing a distinctive role in their pathophysiology [3–5]. In the US, 4 to 6 per 100,000 people are estimated to suffer from MF, with a median survival range from 2 to 11 years [2].

The primary characteristics of MF include bone marrow fibrosis, inadequate or extramedullary hematopoiesis, hepatosplenomegaly, cytopenia, cachexia, and abdominal and constitutional syndromes (fatigue, night sweating, fever) [6, 7]. Literature suggests that the current strategy for managing myelofibrosis (MF) should be guided by risk stratification and prognostication, with an emphasis on a symptom-based approach [1, 8]. Thus, the cornerstone of the MF treatment includes symptom management, patient survival, and improved quality of life (QoL). An improved understanding of the biological mechanisms underlying MPN pathogenesis has contributed to more effective treatment strategies, including interventions aimed at disease modification and enhancing the patient journey [9].

The current MF treatment plan includes Hydroxyurea (anemia-oriented therapy) and JAK inhibitors including Fedratinib, Ruxolitinib, Pacritinib, and Momelotinib, along with other symptomatic drugs [10, 11]. Ruxolitinib was the first drug approved by the United States Food and Drug Administration (US FDA) in 2011 as a first-line treatment option [1]. Reportedly, JAK inhibitors have demonstrated effectiveness in improving overall survival (OS) and QoL of the patients with MF, along with ameliorating the symptom burden [12–17]. Additionally, allogeneic hematopoietic stem cell transplant (HSCT) is also recommended for transplant-eligible high-risk patients with primary MF [18, 19]. Treatment strategies in Indian patients have included Thalidomide, Aspirin, Hydroxyurea, Ruxolitinib, and Prednisolone, with Ruxolitinib generally reserved for cases unresponsive to Hydroxyurea. Despite its approval by the Drug Controller General of India in 2013, access to Ruxolitinib remains limited [20–23].

In line with the Global trend, MF in India results in a significant clinical burden due to symptoms like fatigue, pruritus, night sweating, splenomegaly, microvascular symptoms, and short survival [22]. In India, observational data on MPN patients indicate that PV is the most commonly represented subtype, followed by ET and MF, with cardiovascular events being the leading cause of mortality [20, 21].

Despite significant clinical and economic burden, there is a paucity of data on MF from the Indian context. Further studies are warranted to understand the perception of patients and doctors regarding the severity of the symptoms and the treatment objectives for MF in an Indian context. According to the comprehensive assessment of patient and physician perceptions in different oncology settings, physicians may underestimate the severity and frequency of their patients’ complaints, which might delay or prevent symptom relief [22, 24]. Therefore, it becomes important to bridge the knowledge gap between the MF disease trajectory and the clinical practice, from the real-world perspective. The aim of the present survey was to better understand the MF patient journey and perceptions/practices of Indian hematologists/oncologists regarding diagnostic evaluations, prognostic assessments, and disease management.

## Methodology

### Study design

The current cross-sectional, multicentre, qualitative survey involving patients with MF and their treating physicians was conducted from October 2021 to November 2021 across the four zones (East, West, North, and South) in 17 Indian cities. The survey questionnaire was designed considering the study objectives and reviewed by the investigators (Supplementary Tables 1 and 2). The patient and physician surveys were conducted independently. One-on-one telephonic interviews with the physicians and patients were performed using the Computer-Assisted Personal Interviewing (CAPI) technique. The interviews lasted for no more than 40 min. The MPN-10 score was administered for objective symptom assessment in the patients. The data collected from the physician and patient surveys were primarily related to patient journey, symptomatology, diagnostic attributes, treatment goals and practices, satisfaction with the treatment, in-clinic communication, and patient satisfaction. The differences in physician and patient perceptions throughout the patient journey have been highlighted.

### Study population

A total of 50 physicians including hematologist, medical oncologist, and hemato-oncologist, and 154 patients with MF visiting these physicians were included in the survey.

The physicians were recruited based on telephonic screening by the field staff who targeted haematologists, medical oncologists, and haemato-oncologists across the four zones. Physicians who consented to audio recording and agreed to the follow-up contact for queries were interviewed based on a predefined screening questionnaire. The criteria for screening physicians included the practice type (private or public practice), years in clinical experience (acceptable range: 3–30 years, excluded physicians if experience was less than 3 years or more than 30 years) and number of patients with MF managed by them in the past 12 months (a minimum of 3 patients required). Physicians meeting all criteria proceeded to the main interview, which was scheduled based on their responses.

The selected physicians identified the eligible patients based on the predefined screening criteria. The patients aged > 18 years, diagnosed with MF > 6 months ago, consulted a hematologist, hemato-oncologist, or medical oncologist, and consented to audio recording and re-contact were included. The identified patients were further classified by disease type (primary, post-PV and post-ET MF) and geography (metro vs. non-metro).

### Ethical considerations

The survey was approved by the Royal Pune Independent Ethics Committee, Pune, Maharashtra, India (The Drugs Controller General of India, Registration Number: ECR/45/Indt/MH/2013/RR-19 and RPIEC050921). The research adhered to the guidelines in the Declaration of Helsinki and was conducted according to the Market Research Society’s Code of Conduct. Electronic informed consent was obtained from the survey participants before inclusion in the study. A fair market value honorarium was paid to the survey participants as a token of appreciation for their time invested.

## Results

### Demographics details of the participants

#### Patients

The average age of the study participants was 51 years (22–80 years). The mean age of the patients diagnosed with primary MF was 51 years (range: 22–75 years). The survey had a higher proportion of male patients with MF (68%, *n* = 105). Most of the patients (99%, *n* = 152) in the study belonged to socioeconomic class (SEC) A, defined as the uppermost segment of the consuming class with an average annual household income of INR 7.5 lakhs (USD 8,889) [35]. About 51% (*n* = 78) of the patients in the survey were diagnosed with post-PV myelofibrosis, followed by 33% (*n* = 51) with primary MF, and 16% (*n* = 25) of the patients with post-ET myelofibrosis. The patients were further categorized based on the Dynamic International Prognostic Scoring System (DIPSS) risk stratification at the start of the study, wherein 36% (*n* = 55) of the patients were in the high-risk category followed by intermediate risk-1 (33%, *n* = 50), intermediate risk-2 (26%, *n* = 40) and low risk (8%, *n* = 12). The baseline characteristics of the patients are represented in Table 1.

**Table 1:** Baseline characteristics of patients with myelofibrosis (*N* = 154)

PARAMETERS	PRIMARY MF	POST PV MF	POST ET MF
Age [range]	22–75	25–80	30–80
Age [average]	51	50	51
Gender [ratio]	63:37	71:29	68:32
Males [n]	32	55	17
Females [n]	19	23	8
Calculated prognostic risk score [%, n]			
Low [%, n]	10% [5]	9% [7]	16% [4]
Intermediate-1 [%, n]	39% [20]	37% [29]	28% [7]
Intermediate-2 [%, n]	41% [21]	50% [39]	40% [10]
High [%, n]	10% [5]	4% [3]	16% [4]
Socio-economic class [%, n]			
SEC A [%, n]	100% [51]	97% [76]	100% [25]
SEC B [%, n]	0% [0]	3% [2]	0% [0]
SEC C [%, n]	0% [0]	0% [0]	0% [0]
Annual Household income- Average	**7.89 lacs**	**7.28 lacs**	**7.20 lacs**
Annual Household Income (Range)			
Annual Household Income- <5 lacs [%, n]	37% [19]	42% [33]	40% [10]
Annual Household Income- 5–10 lacs [%, n]	35% [18]	40% [31]	52% [13]
Annual Household Income- >10 lacs [%, n]	27% [14]	18% [14]	8% [2]
Employment status [%, n]			
Corporate executive / Middle level manager	14% [7]	14% [11]	16% [4]
Self-Employed [Own business /shop owner]	41% [21]	42% [33]	44% [11]
Freelancer	16% [8]	9% [7]	0% [0]
Homemaker	20% [10]	22% [17]	28% [7]
Retired	10% [5]	13% [10]	12% [3]
Education status [%, n]			
Uneducated	0% [0]	0% [0]	0% [0]
School up to 4 years	0% [0]	0% [0]	0% [0]
School 5 to 9 years	0% [0]	0% [0]	0% [0]
SSC / HSC	27% [14]	24% [19]	28% [7]
Some College, but not graduate	0% [0]	3% [2]	0% [0]
Graduate / Post-graduate– General	65% [33]	65% [49]	64% [16]
Graduate / Post-graduate– Professional	8% [4]	10% [8]	8% [2]

ET- Essential thrombocythemia; HSC- Higher secondary certificate; MF- Myelofibrosis; n- number of patients; PV- Polycythemia vera, SEC- Socioeconomic class, SSC- Senior secondary certificate

### Physicians

Most physicians (64%, *n* = 32) were hematologists, (20%, *n* = 10) were medical oncologists, and (16%, *n* = 8) were haemato-oncologists, practicing in a private setting (43%, *n* = 21). Average experience of physicians was 16 years (3–30 years) and had consulted an average of 14 patients with MF in the past 12 months.

### Patient journey

The survey explored the participants’ referral and patient journey dynamics. Abdominal pain (81%, *n* = 125), fatigue (77%, *n* = 42), and fever (51%, *n* = 79) were the most experienced symptoms at the time of diagnosis. Additionally, the patients also experienced symptoms like headaches, difficulty sleeping, muscle ache, and bone pain that negatively impacted the QoL. A General practitioner/internal medicine physician was the first point of contact for the patients with MF (58%, *n* = 89), followed by hemato-oncologist/hematologist (39%, *n* = 60) and an oncologist (2%, *n* = 3). After the first point of contact, the majority of patients with MF sought consultation with a second choice of specialists, including hematologists (62%, *n* = 95), hemato-oncologists (24%, *n* = 37), oncologists (13%, *n* = 20), and general physicians (1%, *n* = 2).

An average gap of 10 months (range: 6–24 months) was observed between the onset of symptoms and the final diagnosis. An average time lag of 9 months (range: 6–24 months) was observed between the final diagnosis and the treatment initiation. As per the survey, an average wait-and-watch time of 9 months was reported before initiation of therapy. The patients were mostly treated with Hydroxyurea (55%, *n* = 85), followed by Thalidomide (18%, *n* = 28) and Ruxolitinib (16%, *n* = 25). The remaining patients (10%, *n* = 16) were treated with corticosteroids, androgens, transfusion, and antidepressants. The treatment pattern suggests that Ruxolitinib is the more favoured choice than Thalidomide in Post-PV and post-ET MF, after Hydroxyurea (Table 2**).** The trend observed in the referral dynamics of patients diagnosed with primary MF, post-PV, and post-ET have been represented in Supplementary figure S1.

**Table 2:** Treatment plan in primary MF, Post-PV MF and Post-ET MF patients

Type of MPN	Overall MF		Primary MF		Post-PV MF		Post-ET MF	
MF Treatments	Currently prescribed	Prescribed in the past	Currently prescribed	Prescribed in the past	Currently prescribed	Prescribed in the past	Currently prescribed	Prescribed in the past
Hydroxyurea	55%	54%	49%	49%	55%	54%	64%	64%
Thalidomide	18%	18%	27%	27%	14%	14%	8%	8%
Ruxolitinib	16%	16%	12%	12%	21%	21%	12%	12%

ET- Essential thrombocythemia; MF- Myelofibrosis; MPN- Myeloproliferative neoplasm; PV- Polycythemia vera

In the survey, 66% (*n* = 33) of the physicians stated that they consulted patients with MF monthly, with a similar trend in the metro and non-metro cities (66% and 67%, respectively). However, according to the patients, they have follow-up appointments with their physicians every two months, with intervals ranging from 30 to 90 days.

Additionally, in the survey, 86% (*n* = 132) of patients continued to visit the same doctor. The remaining 14% (*n* = 22) of patients reported a change in their physician; the majority stated they were referred by their previous physician (67%, *n* = 15), relocated to a different area (19%, *n* = 4), or had treatment dissatisfaction (14%, *n* = 3). Apart from the physicians, the patients were also in constant touch with other healthcare professionals, such as nurses (46%) and physician assistants (32%). For most cases the decision-making for the treatment of MF involved both the patient and the physician (556%), but for others it was solely dependent on the physician (36%) or the caregivers alone (8%).

### Risk stratification for MF patients

**Fig. 1:**
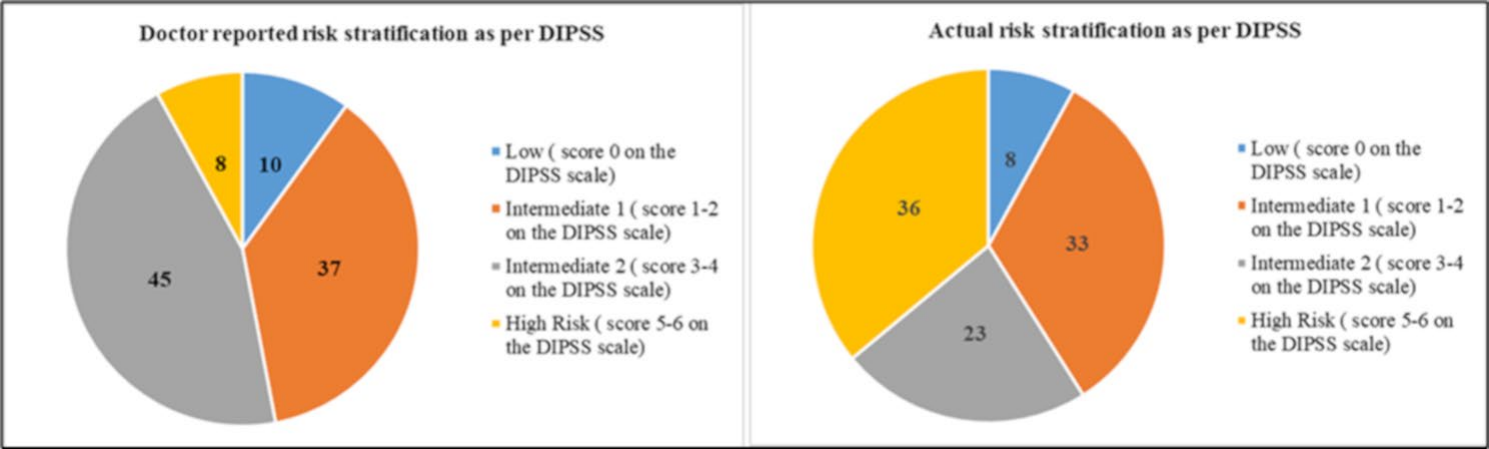
Comparison between actual vs. doctor reported risk stratification as per DIPSS parameters. DIPSS: Dynamic International Prognostic Scoring System

Of the 154 patients with MF, 33% (*n* = 51), 51% (*n* = 78), and 16% (*n* = 25) were diagnosed with primary MF, post-PV, and post-ET, respectively. Most of the physicians (94%, *n* = 47) agreed that they were risk stratifying their patients with MF in the clinic, while remaining 6% of physicians (*n* = 3) did not consider this in their practice. Among the physicians who agreed to risk stratification, 62% (*n* = 31) used the DIPSS system, while others used assessment tools like International Prognostic Scoring System (IPSS) (15%, *n* = 8), Genetically Inspired Prognostic Scoring System (GIPSS) (13%, *n* = 6), Mutation-Enhanced International Prognostic Score System 70+ (MIPSS70) (6%, *n* = 3), and Dynamic International Prognostic Scoring System Plus (DIPSS+) (4%, *n* = 2). Despite 67% (*n* = 103) of the patients being diagnosed as post-PV and post-ET MF, none of the physicians were using the MYSEC-PM in India. According to the risk assessment using DIPSS risk stratification calculation, 36% (*n* = 55) of the patients were classified as high-risk (DIPSS score: 5–6), while 33% (*n* = 18) were classified as intermediate-1 (DIPSS score: 1–2) risk category. Whereas, according to the physician-reported risk stratification, 8% (*n* = 12) of the patients were classified as high-risk (DIPSS score: 5–6), while 45% (*n* = 69) were classified as intermediate-2 (DIPSS score: 3–4) risk category. The exact parameters used for the risk calculation by the physician were not defined to allow for the capture of a natural response. Hence, there was a discordance between the physician estimated risk stratification and patient survey calculated risk (8% vs. 36%) (Fig. 1).

### Treatment goal

The primary treatment goals, as opined by both the physicians and patients, were better QoL (88%, *n* = 44 vs. 74%, *n* = 37), healthy blood counts (66% vs. 40%), and correction of anemia (62% vs. 37%). A smaller number of physicians considered OS (15%, *n* = 8) and symptom improvement (12%, *n* = 6) as treatment goals. In contrast, a considerable number of patients perceived symptom improvement as the primary treatment goal (69%, *n* = 106), and none of the patients’ considered OS as an important treatment outcome (Table 3).

**Table 3:** Treatment goals (Doctors and patients’ response)

Treatment Goals	Doctor Response (*N* = 50) *n* (%)	Patient Response (*N* = 154) *n* (%)
Better quality of life	44 (88%)	114 (74%)
Healthy blood counts	33 (66%)	62 (40%)
Anemia treatment	31 (62%)	57 (37%)
Reduce blood transfusions	14 (27%)	48 (31%)
Reduction in spleen size	11 (21%)	25 (16%)
Overall survival	8 (15%)	NA
Symptom improvement	6 (12%)	106 (69%)
Prevention of vascular/thrombotic events	4 (7%)	28 (18%)
Slow/Delay progression of condition	2 (3%)	23 (15%)

N- Base size; n- number of participants in a specific subgroup

When checking for differences in treatment goals aspired to by physicians in early versus advanced MF, there seems to be no difference in the approach to management, with similar focus on QoL and blood count management, as shown in Table 4.

**Table 4:** Treatment goals in patients with early MF vs. Advanced MF (Physicians response)

Treatment goals	Early MF Patients (*N* = 50) *n* (%)	Advanced MF patients (*N* = 50) *n* (%)
Better quality of life	46 (92%)	42 (84%)
Healthy blood counts	34 (68%)	31 (62%)
Anemia treatment	31 (62%)	31 (62%)
Reduce blood transfusions	14 (28%)	13 (26%)
Reduction in spleen size	9 (18%)	12 (24%)
Overall survival	7 (14%)	8 (16%)
Symptom improvement	4 (8%)	8 (16%)
Prevention of vascular/thrombotic events	3(6%)	4 (8%)
Slow/Delay progression of condition	2 (4%)	1 (2%)

MF- Myelofibrosis; N- Base size; n- number of participants in a specific subgroup

**Fig. 2:**
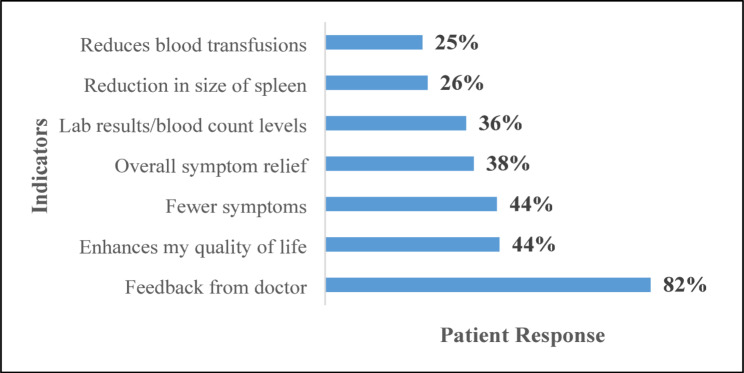
Indicators to determine treatment success (Patients response)

As per the survey, patients perceived positive feedback from physicians (82%), improvement in QoL (44%), and fewer symptoms (44%) as measures to determine the success of MF treatment (Fig. 2).

### Symptom severity assessment

Patients of different risk categories were further stratified into quartiles based on their total symptom score using the MPN-10 symptom assessment tool, where Q1 represented the lowest symptom burden and Q4 represented the highest symptom burden. According to the MPN-10 score, most high-risk patients belonged to Q3 and Q4 (27%, *n* = 15 and 69%, *n* = 38, respectively). Furthermore, in our survey, majority of the low-risk patients (85%, *n* = 11) were in the quartile 4 (Q4) with an MPN-10 score of 79, indicating a high symptom burden despite their low-risk status. In the intermediate-1 risk category, nearly half of the patients are in Q4 (49%, *n* = 25) and Q3 (45%), reflecting a moderate to high symptom burden. Intermediate-2 risk patients are predominantly in Q3 (51%, *n* = 18) and Q4 (43%, *n* = 15), suggesting a high symptom burden as risk increases. When asked about symptom severity assessment in the clinic, 88% (*n* = 44) of physicians claimed to perform symptom severity assessments based on clinical evaluation, and 54% (*n* = 27) of the physicians relied on the MPN-10 questionnaire in clinical practice, while 8% (*n* = 4) assessed symptoms only when activities of daily living were significantly impacted. However, in contrast, the patients opined that only 1% of their doctors were using the MPN-10 questionnaire, which suggests a massive disconnect between the doctors and their patients. Furthermore, we observed an average symptom assessment frequency of 49 days (15–90 days).

### Factors influencing treatment initiation and treatment change decision among physicians

It was observed that 58% (*n* = 29) of physicians-initiated treatment based on the appearance of symptoms, while 42% (*n* = 21) of physicians initiated treatment as soon as MF was diagnosed. Physicians started treatment in symptomatic patients based on the presence of anemia (70%, *n* = 35), abnormal hemoglobin levels (67%, *n* = 34), fever (42%, *n* = 21), unintentional weight loss (38%, *n* = 19), symptomatic splenomegaly (38%, *n* = 19), asymptomatic splenomegaly (36%, *n* = 18), marked leucocytosis (32%, *n* = 16), and day or night sweats (29%, *n* = 15).

The most common reasons for considering changes in drug treatment were changes in symptoms (86%, *n* = 43), changes in blood counts (70%, *n* = 35), cost/insurance coverage of treatments (64%), disease progression (60%, *n* = 30), and lack of efficacy (56%, *n* = 28). The most prominent signs of disease progression for physicians were changes in spleen size, hemoglobin, or any other blood parameters.

### Treatment awareness and satisfaction among patients

In the present survey, patients are aware of the treatment plan, and it aligns with the information provided by the physicians. According to the physicians, 55% (*n* = 85) of the patients were treated with Hydroxyurea, followed by Thalidomide (18%, *n* = 28), and Ruxolitinib (16%, *n* = 25). A small subset of patients (11%, *n* = 16) was treated with antidepressants, androgen corticosteroids, and blood transfusion. The survey reports an average therapy duration of 15 months (5–30 months) for Hydroxyurea, 14 months (5–30 months) for Thalidomide, and 10 months for Ruxolitinib (2–25 months).

Among the prescribed therapies, satisfaction was reported in 19% of patients receiving Hydroxyurea, (*n* = 16), 16% of those on Thalidomide (*n* = 4), and 50% of those on Ruxolitinib (*n* = 13).

Among the 154 patients in the survey, 15 (19%) patients on Hydroxyurea, 11 (7%) patients on Thalidomide, and 8 (5%) patients on Ruxolitinib reported treatment discontinuation. Furthermore, of those who reported therapy discontinuation, half of them cited toxicity as the primary reason, while the other half were unaware of the reason.

## Discussion

This survey conducted among Indian patients with MF and their physicians demonstrates the landscape of MF treatment management in an Indian setting. The survey offers valuable insights into symptom burden, the priority of symptoms requiring immediate resolution, current practices in risk stratification, understanding, and communication on MF, treatment goals, decision-making processes, management of MF, and satisfaction with the current treatment plans from the physician and patient perspective. Notably, the data indicates that the physicians actively involve the patients in the formulation or modification of the treatment plan and regularly assess symptom burden at each visit.

Abdominal pain (81%, *n* = 125), fatigue (77%, *n* = 118), and fever (51%, *n* = 79) were the most common symptoms experienced by patients at the time of diagnosis. Results from various national and international studies report a similar symptom burden. The International Landmark Survey identified fatigue and abdominal pain as common symptoms among the patients with MF [25], while an Indian retrospective review at a tertiary care center reported fatigue, inactivity, and abdominal discomfort as common symptoms in the patients with MF [22].

Our survey found a 10-month gap between symptom onset and final diagnosis, and an additional 9-month delay before initiating treatment. Literature states that early intervention may be more effective than a “watch-and-wait” approach [26]. At the time of the study conduct, the patients were primarily on Hydroxyurea (55%, *n* = 85), Thalidomide (18%, *n* = 28), and Ruxolitinib (16%, *n* = 25), in line with the published studies [24, 25]. A comprehensive evaluation of patient and physician perceptions across various oncology settings reveals that physicians often underestimate the severity and frequency of patients’ concerns, potentially leading to delays or gaps in symptom management and relief [12, 24].

Multiple prognostic risk scoring systems have been developed and used since 2009 [2]. The scoring system helps estimate the OS in patients with MF, thus helping clinicians make better treatment decisions [2, 18, 27]. Physicians managing patients with MF use diverse symptom assessment methods. In alignment with other international findings, our survey revealed that Indian physicians favor the DIPSS (62%, *n* = 31) and the IPSS (15%, *n* = 8) for risk stratification [24, 28]. The survey findings reveal a discordance between the physician-estimated risk stratification and calculated risk from patient survey parameters (8% vs. 36%). The discrepancy between survey results and physician-assigned scores may suggest differences in perception between real-world physician experiences and the actual risk calculation framework. It can be argued that lack of adherence to DIPSS score by the physicians in routine clinical practice could cause underestimation of high-risk patients and lead to delayed treatment initiation.

In our survey, 88% of the physicians (*n* = 44) relied on clinical evaluation, and 54% (*n* = 27) used the MPN-10 questionnaire. As recorded by the MPN-10 system, among all risk categories, the highest patient proportion was present in Q3 and Q4. The Q4 represented most of the low-risk (85%, *n* = 11/13), intermediate-1 risk (49%, *n* = 25/51), and high-risk (69%, *n* = 38/55) patients, whereas Q3 represented the majority of intermediate-2 risk (51%, *n* = 18/35) patients. This was in line with the findings from other MPN surveys [24, 25, 29]. Based on our study results, it is evident that the MPN-10 score assessment is a crucial tool for evaluating symptom burden in patients with MPNs.

The DIPSS provides valuable risk stratification, but the MPN-10 score offers an essential layer of insight into the symptom burden experienced by patients across all risk categories. As per our survey outcomes, physicians must utilize both DIPSS and the MPN-10 score to achieve a more holistic understanding of patient needs, facilitating better-targeted interventions and improving overall patient outcomes.

The survey findings suggests that the physicians preferred Hydroxyurea (86%, *n* = 43), transfusion (82%, *n* = 41), and Ruxolitinib (78%, *n* = 39) as the primary treatment options in patients with MF. Both physicians and patients considered better QoL as the primary treatment goal. Physicians rely on laboratory investigations (anemia and healthy blood count) to assess improvements. However, patients’ self-assessment of improvement was more subjective, focusing on overall symptom improvement and its positive impact on their daily life. Our findings were mostly aligned with the US Landmark [30] and the International Landmark survey [25], while the Taiwan Landmark survey [24] observed a fair agreement between the physicians and patients in treatment goals and mutual communication regarding MPN. Additionally, less frequent consultation (once every two months) may reduce treatment satisfaction and adherence.

Symptom burden drives treatment initiation. Spleen size, anemia, fever, and abnormal blood counts were the most common factors considered by the physicians. The appearance of new symptoms and increased blood counts were the main causes for the change in therapy. However, in our survey, the patients reported many symptoms with severity beyond the ones considered by the physicians, such as bone pain, difficulty in sleeping, sexual problems, depression, and inactivity. Current guidelines recommend a personalized approach considering risk, spleen size, phenotype, and patient preferences [14, 30–33]. Thus, as established in other studies, the therapies in our survey also help reduce the symptom burden and increase the OS [25, 30].

Despite challenges, patients in our survey were aware of their treatments and reported satisfaction with Hydroxyurea (*n* = 16), Ruxolitinib (*n* = 13), and Thalidomide (*n* = 4). The COMFORT-I trial established role of Ruxolitinib in overall symptom score improvement, including bone pain and inactivity (Supplementary Figure S2) [34]. The COMFORT-II trial (Ruxolitinib vs. best available therapy) also showed significant and durable reduction in MF-related symptoms, splenomegaly, and improvement in the overall QoL with modest toxic effects in the Ruxolitinib group at week 48 (Supplementary Figure S3) [35]. Passamonti F et al. in their study demonstrated longer OS compared to conventional therapy [36]. The JAKoMo and JUMP trials further confirmed its long-lasting symptom relief and manageable safety profile, demonstrating Ruxolitinib’s pivotal role in MF management [37, 38]. Thus, the results from various studies highlight the role of Ruxolitinib in improving the overall QoL and symptom burden in patients with MF.

### Limitations

The survey is associated with selection bias since the physicians were selected by certain qualifying criteria and their willingness to participate in this study. Physicians only recruited patients who had a certain level of education and/or financial resources, allowing them to understand and complete the survey. Hence, the results obtained may not be generalized to the overall population of patients with MF in India. Furthermore, since the patients were identified by the physicians from their patient pool, the possibility of bias in the patient’s responses exist. Most of the physicians surveyed were from the private sector, with minimal representation from government settings. The survey does not explore a detailed analysis of epidemiological variation or historical and current medical history. Patients’ self-reporting might also lead to inconsistencies and bias. The impact of various therapies on the OS was not determined in the survey.

## Conclusion

In summary, the survey strengthens the importance of understanding symptom burden, perceptions of symptomatology, and key factors influencing treatment decisions. The findings highlight the growing need for a comprehensive approach that uses both DIPSS and MPN-10 assessment tools for risk stratification and symptom severity evaluation. Bridging the gap between physicians and patients requires enhanced patient education on disease progression, treatment outcomes and a shared understanding of treatment objectives. Physicians need to be more proactive in explaining the actual disease condition to the patient and its progression so that the treatment expectations can be aligned better. Better emotional connection with the patients, regular consultation with appropriate time during the follow-up visits, and proactively explaining their condition may help in better treatment compliance.

## Electronic supplementary material

Below is the link to the electronic supplementary material.

Supplementary Material 1

## Data Availability

Individually deidentified participant data will be made available on reasonable request, starting from the date of publication, until 6 months after publication. Requests beyond this time frame will be considered on a case-by-case basis. Requests for data should be directed to Dr Disha Shetty, disha.shetty@novartis.com.
