# Supplementary material for: A comprehensive overview of patient journey and management decision pathway for myelofibrosis in India: INLAND survey
Source: BMC Cancer. 2025 Jul 10;25:1164. doi: 10.1186/s12885-025-14476-3 (PMC12247463; doi:10.1186/s12885-025-14476-3)

**SUPPLEMENTARY FILE**

# Supplementary Table 1: The physician questionnaire

| **SECTION I: PHYSICIAN DEMOGRAPHICS AND CASELOAD** |
| --- |

1. Does your center have a stem cell transplant facility? **SELECT ANY ONE OPTION**

| **YES** | **NO** |
| --- | --- |

2A. Has your clinic/practice ever participated as a study site for Myelofibrosis clinical trials? **SELECT ANY ONE OPTION**

| **YES** | **NO** |
| --- | --- |

***ASK ONLY IF THE RESPONDENT HAS CODED “YES” IN Q2A, ELSE SKIP TO Q2C***

2B. How many Myelofibrosis clinical trials has your clinic been involved in the last 2 years? **PLEASE ENTER A NUMBER**

*Programming Instructions: Range for this response is 0-100*

|  |
| --- |

2C. Based on your overall practice, what percent do you estimate is made up of a) Hematology cases and b) Myelofibrosis cases & c) any others? **PLEASE ENTER A NUMBER FOR ALL 3. TOTAL TO ADD UP TO 100%.**

| 1. **HAEMATOLOGY CASES** | % |
| --- | --- |
| 1. **MYELOFIBROSIS CASES** | % |
| 1. **OTHERS (PLEASE SPECIFY)** | % |
| **TOTAL** | **100%** |

2D. Out of 100 Myelofibrosis patients at diagnosis, what proportion of patients would be classified under each of the severity groups mentioned below? **PLEASE ENTER A NUMBER FOR EACH SEVERITY. TOTAL TO ADD UP TO 100%**

| **SEVERITY OF SYMPTOMS** | **PROPORTION OF MYELOFIBROSIS PATIENTS**  ***(At the time of diagnosis)*** |
| --- | --- |
| No Symptoms | % |
| Mild to no Symptoms | % |
| Moderate symptoms | % |
| Severe symptoms | % |
| **TOTAL** | **100%** |

2E. Out of 100%, what proportion of all your patients that are currently under follow up have? **MENTION PROPORTION FOR EACH CATEGORY OF SYMPTOMS. TOTAL TO ADD UP TO 100%**

| **SEVERITY OF SYMPTOMS** | **PROPORTION OF MYELOFIBROSIS PATIENTS**  ***(In current practice)*** |
| --- | --- |
| No Symptoms | % |
| Mild to no Symptoms | % |
| Moderate symptoms | % |
| Severe symptoms | % |
| **TOTAL** | **100%** |

| **SECTION II: PATIENT DISEASE BURDEN** |
| --- |

1. Doctor assuming 10 Myelofibrosis patients, at the time of diagnosis, what proportion of your patients do you choose to observe instead of recommending drug treatment?  **MENTION PROPORTION.** **PLEASE ENTER A NUMBER**

*Programming Instructions: Range for this response is 0-100*

|  | **PROPORTION OF MYELOFIBROSIS PATIENTS OBSERVED INSTEAD OF RECOMMENDING TREATMENT** |
| --- | --- |

1. What are the 5 symptoms you most often (%) hear about from your patients for Myelofibrosis? **SELECT ALL THAT APPLY**

*Programming instructions: Respondent should be allowed to type exactly 5 responses and an intuitive list of the below options should be presented as the respondent types in first 2-3 letters*

| **SYMPTOM MOST OFTEN HEARD FROM MYELOFIBROSIS PATIENTS** | **CODE** |
| --- | --- |
| Abdominal discomfort | 1 |
| Abdominal pain | 2 |
| Bone pain (diffuse/not joint pain or arthritis) | 3 |
| Bruising | 4 |
| Cough | 5 |
| Depression or sad mood | 6 |
| Difficulty Sleeping | 7 |
| Dizziness/Vertigo/Light-headedness | 8 |
| Facial flushing | 9 |
| Fatigue or tiredness | 10 |
| Feeling of fullness quickly when eating (early satiety) | 11 |
| Fever | 12 |
| Headaches | 13 |
| Inactivity | 14 |
| Increased daytime sweating | 15 |
| Itching (pruritus) | 16 |
| Muscle aches | 17 |
| Night sweats | 18 |
| Nosebleeds | 19 |
| Numbness/Tingling in hands and feet | 20 |
| Problems with concentration | 21 |
| Problems with sexual desire or function | 22 |
| Redness, throbbing and burning pain in the hands and feet | 23 |
| Shortness of breath | 24 |
| Unintentional weight loss | 25 |
| Weakness | 26 |
| *Others (please specify)* | *99* |

1. Doctor, from the top 5 symptoms for Myelofibrosis that you have mentioned, can you please rate each of those symptoms based on their negative effect on the patients quality of life on a scale of 1 to 10, where 1 means “NOT AT ALL” and 10 means “A GREAT DEAL”? **GIVE A RATING BETWEEN 1-10 FOR EACH SYMPTOM**

*Programming Instructions: Link to display only those 5 symptoms mentioned by the respondent in Q4*

| **NOT AT ALL** |  |  |  |  |  |  |  |  | **A GREAT DEAL** |
| --- | --- | --- | --- | --- | --- | --- | --- | --- | --- |
| 1 | 2 | 3 | 4 | 5 | 6 | 7 | 8 | 9 | 10 |

| **NEGATIVE IMPACT OF MYELOFIBROSIS SYMPTOMS ON QUALITY OF LIFE** | **NOT AT ALL** |  |  |  |  |  |  |  |  | **A GREAT DEAL** |
| --- | --- | --- | --- | --- | --- | --- | --- | --- | --- | --- |
| Abdominal discomfort | 1 | 2 | 3 | 4 | 5 | 6 | 7 | 8 | 9 | 10 |
| Abdominal pain | 1 | 2 | 3 | 4 | 5 | 6 | 7 | 8 | 9 | 10 |
| Bone pain (diffuse/not joint pain or arthritis) | 1 | 2 | 3 | 4 | 5 | 6 | 7 | 8 | 9 | 10 |
| Bruising | 1 | 2 | 3 | 4 | 5 | 6 | 7 | 8 | 9 | 10 |
| Cough | 1 | 2 | 3 | 4 | 5 | 6 | 7 | 8 | 9 | 10 |
| Depression or sad mood | 1 | 2 | 3 | 4 | 5 | 6 | 7 | 8 | 9 | 10 |
| Difficulty Sleeping | 1 | 2 | 3 | 4 | 5 | 6 | 7 | 8 | 9 | 10 |
| Dizziness/Vertigo/Light-headedness | 1 | 2 | 3 | 4 | 5 | 6 | 7 | 8 | 9 | 10 |
| Facial flushing | 1 | 2 | 3 | 4 | 5 | 6 | 7 | 8 | 9 | 10 |
| Fatigue or tiredness | 1 | 2 | 3 | 4 | 5 | 6 | 7 | 8 | 9 | 10 |
| Feeling of fullness quickly when eating (early satiety) | 1 | 2 | 3 | 4 | 5 | 6 | 7 | 8 | 9 | 10 |
| Fever | 1 | 2 | 3 | 4 | 5 | 6 | 7 | 8 | 9 | 10 |
| Headaches | 1 | 2 | 3 | 4 | 5 | 6 | 7 | 8 | 9 | 10 |
| Inactivity | 1 | 2 | 3 | 4 | 5 | 6 | 7 | 8 | 9 | 10 |
| Increased daytime sweating | 1 | 2 | 3 | 4 | 5 | 6 | 7 | 8 | 9 | 10 |
| Itching (pruritus) | 1 | 2 | 3 | 4 | 5 | 6 | 7 | 8 | 9 | 10 |
| Muscle aches | 1 | 2 | 3 | 4 | 5 | 6 | 7 | 8 | 9 | 10 |
| Night sweats | 1 | 2 | 3 | 4 | 5 | 6 | 7 | 8 | 9 | 10 |
| Nosebleeds | 1 | 2 | 3 | 4 | 5 | 6 | 7 | 8 | 9 | 10 |
| Numbness/Tingling in hands and feet | 1 | 2 | 3 | 4 | 5 | 6 | 7 | 8 | 9 | 10 |
| Problems with concentration | 1 | 2 | 3 | 4 | 5 | 6 | 7 | 8 | 9 | 10 |
| Problems with sexual desire or function | 1 | 2 | 3 | 4 | 5 | 6 | 7 | 8 | 9 | 10 |
| Redness, throbbing and burning pain in the hands and feet | 1 | 2 | 3 | 4 | 5 | 6 | 7 | 8 | 9 | 10 |
| Shortness of breath | 1 | 2 | 3 | 4 | 5 | 6 | 7 | 8 | 9 | 10 |
| Unintentional weight loss | 1 | 2 | 3 | 4 | 5 | 6 | 7 | 8 | 9 | 10 |
| Weakness | 1 | 2 | 3 | 4 | 5 | 6 | 7 | 8 | 9 | 10 |
| *Others (please specify)* | *1* | *2* | *3* | *4* | 5 | 6 | 7 | 8 | 9 | 10 |

1. Out of all the symptoms that patients experience with their Myelofibrosis, which three symptoms do you perceive they would most want to resolve? **PLEASE ASSIGN RANKING FROM 1-3 FOR 3 SYMPTOMS WITH RANK 1 BEING THE MOST IMPORTANT AND RANK 3 BEING THE LEAST IMPORTANT**

*Programming Instructions: Link to display only those 5 symptoms mentioned by the respondent in Q4. Respondent should be allowed to select only 3 options and rank them as 1^st^, 2^nd^ and 3^rd^.*

| **SYMPTOMS THAT PATIENTS WANT TO RESOLVE THE MOST** | **RANK** | | |
| --- | --- | --- | --- |
| Abdominal discomfort | 1 | 2 | 3 |
| Abdominal pain | 1 | 2 | 3 |
| Bone pain (diffuse/not joint pain or arthritis) | 1 | 2 | 3 |
| Bruising | 1 | 2 | 3 |
| Cough | 1 | 2 | 3 |
| Depression or sad mood | 1 | 2 | 3 |
| Difficulty Sleeping | 1 | 2 | 3 |
| Dizziness/Vertigo/Light-headedness | 1 | 2 | 3 |
| Facial flushing | 1 | 2 | 3 |
| Fatigue or tiredness | 1 | 2 | 3 |
| Feeling of fullness quickly when eating (early satiety) | 1 | 2 | 3 |
| Fever | 1 | 2 | 3 |
| Headaches | 1 | 2 | 3 |
| Inactivity | 1 | 2 | 3 |
| Increased daytime sweating | 1 | 2 | 3 |
| Itching (pruritus) | 1 | 2 | 3 |
| Muscle aches | 1 | 2 | 3 |
| Night sweats | 1 | 2 | 3 |
| Nosebleeds | 1 | 2 | 3 |
| Numbness/Tingling in hands and feet | 1 | 2 | 3 |
| Problems with concentration | 1 | 2 | 3 |
| Problems with sexual desire or function | 1 | 2 | 3 |
| Redness, throbbing and burning pain in the hands and feet | 1 | 2 | 3 |
| Shortness of breath | 1 | 2 | 3 |
| Unintentional weight loss | 1 | 2 | 3 |
| Weakness | 1 | 2 | 3 |
| *Others (please specify)* | *1* | *2* | *3* |

1. Considering all the myelofibrosis patients you have treated in the past 6 months, what is the impact of each of these experiences/feelings on their quality of life, because of myelofibrosis alone and not because of their treatment? Please let us know the impact on a scale of 1 to 10, where 1 means “NOT AT ALL” and 10 means “A GREAT DEAL”? **PLEASE ASSIGN A RATING BETWEEN 1-10 FOR EACH STATEMENT**

| **NOT AT ALL** |  |  |  |  |  |  |  |  | **A GREAT DEAL** |
| --- | --- | --- | --- | --- | --- | --- | --- | --- | --- |
| 1 | 2 | 3 | 4 | 5 | 6 | 7 | 8 | 9 | 10 |

| **EXPERIENCE OF MYELOFIBROSIS PATIENT** | **NOT AT ALL** |  |  |  |  |  |  |  |  | **A GREAT DEAL** |
| --- | --- | --- | --- | --- | --- | --- | --- | --- | --- | --- |
| Irritable or unusually angry and I have not controlled it well | 1 | 2 | 3 | 4 | 5 | 6 | 7 | 8 | 9 | 10 |
| Their condition has caused emotional hardship for them | 1 | 2 | 3 | 4 | 5 | 6 | 7 | 8 | 9 | 10 |
| Their condition has caused financial hardship for them | 1 | 2 | 3 | 4 | 5 | 6 | 7 | 8 | 9 | 10 |
| Their condition has caused physical hardship for them | 1 | 2 | 3 | 4 | 5 | 6 | 7 | 8 | 9 | 10 |
| Their condition is controlling their life | 1 | 2 | 3 | 4 | 5 | 6 | 7 | 8 | 9 | 10 |
| Their sleeping habits have negatively changed | 1 | 2 | 3 | 4 | 5 | 6 | 7 | 8 | 9 | 10 |
| They feel embarrassed because of their physical appearance due to their disease | 1 | 2 | 3 | 4 | 5 | 6 | 7 | 8 | 9 | 10 |
| They have felt depressed | 1 | 2 | 3 | 4 | 5 | 6 | 7 | 8 | 9 | 10 |
| They have felt discouraged | 1 | 2 | 3 | 4 | 5 | 6 | 7 | 8 | 9 | 10 |
| They have noticed a change in their appetite | 1 | 2 | 3 | 4 | 5 | 6 | 7 | 8 | 9 | 10 |
| Trouble coping with the stress they’ve been having | 1 | 2 | 3 | 4 | 5 | 6 | 7 | 8 | 9 | 10 |

1. Overall, to what extent does their myelofibrosis negatively impact a patient’s activities of daily living on a scale of 1-10 where 1 means “NOT AT ALL” and 10 means “A GREAT DEAL”? **PLEASE ASSIGN A RATING BETWEEN 1-10 FOR THE EXTENT OF NEGATIVE IMPACT OF MYELOFIBROSIS**

| **OVERALL NEGATIVE IMPACT OF MYELOFIBROSIS** | **NOT AT ALL** |  |  |  |  |  |  |  |  | **A GREAT DEAL** |
| --- | --- | --- | --- | --- | --- | --- | --- | --- | --- | --- |
|  | 1 | 2 | 3 | 4 | 5 | 6 | 7 | 8 | 9 | 10 |

1. To what extent do you feel that your patient’s condition interferes with the following activities in their lives on a scale of 1 to 10, where 1 means “NOT AT ALL” and 10 means “A GREAT DEAL”? **PLEASE ASSIGN A RATING BETWEEN 1-10 FOR EACH OPTION**

| **NOT AT ALL** |  |  |  |  |  |  |  |  | **A GREAT DEAL** |
| --- | --- | --- | --- | --- | --- | --- | --- | --- | --- |
| 1 | 2 | 3 | 4 | 5 | 6 | 7 | 8 | 9 | 10 |

| **EXPERIENCE OF MYELOFIBROSIS PATIENT** | **NOT AT ALL** |  |  |  |  |  |  |  |  | **A GREAT DEAL** |
| --- | --- | --- | --- | --- | --- | --- | --- | --- | --- | --- |
| Pain and discomfort have caused them to limit their activities | 1 | 2 | 3 | 4 | 5 | 6 | 7 | 8 | 9 | 10 |
| Their condition has interfered with their daily activities | 1 | 2 | 3 | 4 | 5 | 6 | 7 | 8 | 9 | 10 |
| Their condition has interfered with their family or social life | 1 | 2 | 3 | 4 | 5 | 6 | 7 | 8 | 9 | 10 |
| Their condition has interfered with their relationship with their caregiver | 1 | 2 | 3 | 4 | 5 | 6 | 7 | 8 | 9 | 10 |
| Even mild to moderate symptoms can have a negative impact on the quality of life for patients with Myelofibrosis | 1 | 2 | 3 | 4 | 5 | 6 | 7 | 8 | 9 | 10 |
| A Myelofibrosis patients QoL is significantly affected in case of severe splenomegaly | 1 | 2 | 3 | 4 | 5 | 6 | 7 | 8 | 9 | 10 |
| A Myelofibrosis patients QoL is not significantly affected in case of severe splenomegaly | 1 | 2 | 3 | 4 | 5 | 6 | 7 | 8 | 9 | 10 |
| Their condition has interfered with their sex life | 1 | 2 | 3 | 4 | 5 | 6 | 7 | 8 | 9 | 10 |

| **SECTION III: PATIENT MANAGEMENT AND TREATMENT DECISIONS** |
| --- |

1. Do you classify your Myelofibrosis patients according to prognostic risk category? **SELECT ANY ONE OPTION**

| **YES** | **NO** |
| --- | --- |

***ASK ONLY THOSE RESPONDENTS THAT CODE “YES” IN Q10***

1. Which prognostic assessment system do you utilize the most in your current practice? **SELECT ANY ONE OPTION**

| **PROGNOSITIC ASSESSMENT SYSTEM** | **CODE** |
| --- | --- |
| Age Adjusted Dynamic International Prognostic Scoring System (aaDIPSS) | 1 |
| Dynamic International Prognostic Scoring System (DIPSS) | 2 |
| Dynamic International Prognostic Scoring System Plus (DIPSS+) | 4 |
| Genetically Inspired Prognostic Scoring System (GIPSS) | 5 |
| International Prognostic Scoring System (IPSS) | 6 |
| Mutation-Enhanced International Prognostic Score System 70+ (MIPSS70) | 7 |
| Myelofibrosis Secondary to PV and ET Prognostic Model (MYSEC- PM) | 8 |
| *Any others (please specify)* | *99* |

***ASK ONLY THOSE RESPONDENTS THAT CODE “NO” IN Q10***

1. Please select the most appropriate reason for not prognosticating the patient **SELECT ALL THAT APPLY**

| **REASONS FOR NOT USING PROGNOSTIC ASSESMENTS SYSTEMS** | **CODE** |
| --- | --- |
| I am not familiar with the prognostic assessments available | 1 |
| I am familiar but do not find the prognostic assessments useful | 2 |
| I am familiar and think they are useful but do not have the time to make individual assessments | 3 |
| *Others (please specify)* | *99* |

***ASK ALL RESPONDENTS***

1. Which of the following best describes how you assess a Myelofibrosis patient’s symptoms during a routine patient visit? **SELECT ANY ONE OPTION**

| **DISCUSSION DURING CONSULTATION** | **CODE** |
| --- | --- |
| They listen and wait for me to tell them about any symptoms | 1 |
| I proactively ask them about symptoms or how they are feeling | 2 |
| Specifically tell them about most important symptoms | 3 |
| Ask them to fill a symptom check list and then review each symptom | 4 |
| Do not talk to them about any symptoms | 5 |
| They do not show any interest in the symptoms mentioned | 6 |
| *Others (please specify)* | *99* |

1. Which of the following best describes how you discuss the disease and prognosis with your Myelofibrosis patients during an average patient counselling? **PLEASE SELECT ALL THAT APPLY**

| **SYMPTOMS DURING CONSULTATION** | **CODE** |
| --- | --- |
| The disease and management are discussed as a cancer | 1 |
| The disease is discussed as a condition that may progress to cancer | 2 |
| It is discussed as a blood disorder that can be managed | 3 |
| It is discussed as a condition that needs life-long treatment | 4 |
| Approached as condition requiring symptomatic care | 5 |
| I don’t discuss symptoms with my patients | 6 |
| *Others (please specify)* | *99* |

1. A. Considering all your MF patients, could you let us know the frequency of a complete assessment of symptom presence or severity? **SELECT ANY ONE OPTION**

B. Considering all your MF patients, could you let us know the frequency that you suggest for your patients to follow up with you? **SELECT ANY ONE OPTION**

| **FREQUENCY OF SYMPTOM SEVERITY ASSESSMENT** | **FREQUENCY OF FOLLOW UP WITH PATIENTS** | **CODE** |
| --- | --- | --- |
| Every 15 days | Every 15 days | 1 |
| Every month | Every month | 2 |
| Once every 2 months | Once every 2 months | 3 |
| Once every 3 months | Once every 3 months | 4 |
| Once every 6 months | Once every 6 months | 5 |
| Once per year | Once per year | 6 |
| *Others (please specify)* | *Others (please specify)* | *99* |

1. What tool(s) or approaches do you use to assess symptom severity in your Myelofibrosis patients? **SELECT ALL THAT APPLY**

| **TOOLS / APPROACHES FOR SYMPTOM ASSESSMENT** | **CODE** |
| --- | --- |
| MPN-10 Questionnaire | 1 |
| On your own rating | 2 |
| Significance of impact on their activities of daily living | 3 |
| *Others (please specify)* | *99* |

1. What proportion of your Myelofibrosis patients recognize their symptoms as being related to Myelofibrosis? **SELECT ANY ONE OPTION**

| **PROPORTION OF PATIENT RECOGNIZING MYELOFIBROSIS SYMPTOMS** | **CODE** |
| --- | --- |
| None | 1 |
| Few | 2 |
| Some | 3 |
| Almost all | 4 |
| All | 5 |
| I don’t know | 6 |

1. What do you look for when assessing disease progression in your patients? **SELECT ALL THAT APPLY**

| **FACTORS CONSIDERED FOR MONITORING DISEASE PROGRESSION** | **CODE** |
| --- | --- |
| Blood smear examination for abnormal cells | 1 |
| Change in hemoglobin | 2 |
| Change in platelets | 3 |
| Change in spleen size | 4 |
| Change in status or severity of symptoms in patients under observation | 5 |
| Change in transfusion status / frequency | 6 |
| Change in white blood cell counts | 7 |
| Hydroxyurea dose increase | 8 |
| Increasing blasts | 9 |
| Increasing weight loss | 10 |
| Presence of a new symptom | 11 |
| Worsening of disease signs and symptoms on current therapy | 12 |
| *Others (please specify)* | *99* |

1. A. In your Myelofibrosis patients, do you ever begin treatment based on their symptoms alone? **SELECT ANY ONE OPTION**

| **YES** | **NO** |
| --- | --- |

***ASK Q19B ONLY TO THOSE RESPONDENTS WHO HAVE SELECTED “YES” IN Q19A***

1. What proportion of those patients, who are experiencing any of the below, do you ever recommend drug treatment? **PLEASE ASSIGN PROPORTIONS FOR EACH FACTOR. TOTAL CAN BE BELOW / EXCEED 100%**

| **FACTORS CONSIDERED FOR DRUG TREATMENT** | **PROPORTION** |
| --- | --- |
| Abnormal Hemoglobin levels | % |
| Anemia | % |
| Asymptomatic splenomegaly | % |
| Day or night sweats | % |
| Fever | % |
| Marked Leukocytosis | % |
| Symptomatic Splenomegaly | % |
| Unintentional Weight Loss | % |

***ASK ALL RESPONDENTS***

1. Which of the following treatments have you ever prescribed for your Myelofibrosis patients? **SELECT ALL THAT APPLY**
2. Which of the following treatments are prescribed to your current Myelofibrosis patients? **SELECT ALL THAT APPLY**

***ONLY DISPLAY THOSE TREATMENTS THAT THE RESPONDENT HAS CODED IN Q20. MULTIPLE CODING***

| **MYELOFIBROSIS TREATMENTS** | **EVER PRESCRIBED** | **CURRENTLY PRESCRIBING** |
| --- | --- | --- |
| Alternative Treatments | 1 | 1 |
| Androgens | 2 | 2 |
| Antidepressants | 3 | 3 |
| Antihistamines | 4 | 4 |
| Bone marrow transplant or stem cell transplant | 5 | 5 |
| Corticosteroids | 6 | 6 |
| Counselling | 7 | 7 |
| Epoetin alfa injection | 8 | 8 |
| Hydroxyurea | 9 | 9 |
| Interferon (e.g. IFN-α) | 10 | 10 |
| Investigational drug in the context of a clinical trial | 11 | 11 |
| Iron replacement therapies | 12 | 12 |
| Lenalidomide | 13 | 13 |
| Radiation therapy | 14 | 14 |
| Ruxolitinib | 15 | 15 |
| Splenectomy (removal of spleen) | 16 | 16 |
| Thalidomide | 17 | 17 |
| Transfusion | 18 | 18 |
| None of the above | 19 | 19 |
| *Others (please specify)* | *99* | *99* |

***ASK ONLY THOSE RESPONDENTS WHO HAVE CODED “5” IN Q20 OR Q21***

1. A. Is HSCT a major intervention for Myelofibrosis patients in your practice? **SELECT ANY ONE OPTION**

| **YES** | **NO** |
| --- | --- |

***ASK ONLY THOSE RESPONDENTS WHO HAVE CODED “5” IN Q20 OR Q21***

B. Out of every 10 Myelofibrosis patients how many can undergo transplant in your practice? **PLEASE ENTER A NUMBER**

*Programming instructions: Range of response for every category of HCP is 0-10*

|  | **NUMBER OF PATIENTS THAT ARE ELIGIBLE FOR TRANSPLANT** |
| --- | --- |

1. A. In your Early-stage MF patients, other than a cure, what are your most important treatment goals for their therapy?

*For this question consider a patient having no to <5 cms spleen, fatigue and bone pain, adequate cell counts OR low to Int-1 risk on IPSS/DIPSS, not eligible for a transplant*

**PLEASE ASSIGN RANKING FROM 1-3 FOR TOP 3 TREATMENT GOALS WITH RANK 1 BEING THE MOST IMPORTANT AND RANK 3 BEING THE LEAST IMPORTANT**

B. In your Late-stage MF patients, other than a cure, what are your most important treatment goals for their therapy?

*For this question consider a patient with Spleen >5 cms, with night sweats and fever, cytopenia on 1 or more cell lines OR int-2 or high-risk MF on IPSS/DIPSS, not eligible for transplant*

**PLEASE ASSIGN RANKING FROM 1-3 FOR TOP 3 TREATMENT GOALS WITH RANK 1 BEING THE MOST IMPORTANT AND RANK 3 BEING THE LEAST IMPORTANT**

| **TREATMENT GOALS IN MYELOFIBROSIS** | **RANK- Q23A**  **EARLY MF PATIENTS** | | | **RANK- Q23B**  **ADVANCED MF PATIENTS** | | |
| --- | --- | --- | --- | --- | --- | --- |
| Anemia treatment | 1 | 2 | 3 | 1 | 2 | 3 |
| Better quality of life | 1 | 2 | 3 | 1 | 2 | 3 |
| Healthy blood counts | 1 | 2 | 3 | 1 | 2 | 3 |
| Prevention of vascular/thrombotic events | 1 | 2 | 3 | 1 | 2 | 3 |
| Reduce blood transfusions | 1 | 2 | 3 | 1 | 2 | 3 |
| Reduction in spleen size | 1 | 2 | 3 | 1 | 2 | 3 |
| Slow/Delay progression of condition | 1 | 2 | 3 | 1 | 2 | 3 |
| Symptom improvement | 1 | 2 | 3 | 1 | 2 | 3 |
| Overall survival | 1 | 2 | 3 | 1 | 2 | 3 |
| Does not need treatment at this stage | 1 | 2 | 3 |  |  |  |
| *Others (please specify)* | *1* | *2* | *3* | *1* | *2* | *3* |

1. In which of the below circumstances would you change drug therapy for your Myelofibrosis patients? **SELECT ALL THAT APPLY**

*Respondent should be allowed to select only 5 options and rank them as 1^st^, 2^nd^, 3^rd^, 4^th^ & 5^th^*

| **CIRCUMSTANCES FOR CHANGE IN DRUG THERAPY** | **RANK** | | | | |
| --- | --- | --- | --- | --- | --- |
| Change of symptoms | 1 | 2 | 3 | 4 | 5 |
| Cost/coverage | 1 | 2 | 3 | 4 | 5 |
| Cytopenia | 1 | 2 | 3 | 4 | 5 |
| Disease progression | 1 | 2 | 3 | 4 | 5 |
| Increase in blood counts | 1 | 2 | 3 | 4 | 5 |
| Lack of efficacy | 1 | 2 | 3 | 4 | 5 |
| Patient preference | 1 | 2 | 3 | 4 | 5 |
| Side effects | 1 | 2 | 3 | 4 | 5 |
| *Others (please specify)* | 1 | 2 | 3 | 4 | 5 |

| **SECTION IV: PHYSICIAN PERCEPTIONS** |
| --- |

1. On a scale from 1 (not at all) to 10 (a great deal), to what extent do your Myelofibrosis patients want to be involved in decisions regarding their treatment? **PLEASE ASSIGN RATING BETWEEN 1-10 FOR PATIENT INVOLVEMENT OF PATIENTS IN TREATMENT DECISIONS**

| **INVOLVEMENT IN TREATMENT DECISIONS** | **NOT AT ALL** |  |  |  |  |  |  |  |  | **A GREAT DEAL** |
| --- | --- | --- | --- | --- | --- | --- | --- | --- | --- | --- |
|  | 1 | 2 | 3 | 4 | 5 | 6 | 7 | 8 | 9 | 10 |

1. How often does a Myelofibrosis patient not agree with your primary treatment recommendation? **SELECT ONLY ONE OPTION**

| **AGREEMENT WITH TREATMENT RECOMMENDATIONS** | **CODE** |
| --- | --- |
| Never | 1 |
| Sometimes | 2 |
| Often | 3 |
| Always | 4 |

1. In general, what are your satisfaction levels with the individual **symptom management** of your Myelofibrosis patients? **SELECT ANY ONE OPTION**

| **SATISFACTION WITH SYMPTOM MANAGEMENT PRACTICES** | **CODE** |
| --- | --- |
| Very dissatisfied | 1 |
| Somewhat dissatisfied | 2 |
| Somewhat satisfied | 3 |
| Very satisfied | 4 |
| *Don’t know / Can’t say* | *99* |

1. In general, what is the satisfaction level of your Myelofibrosis patients with the **overall treatment and management** of their condition? **SELECT ANY ONE OPTION**

| **OVERALL SATISFACTION WITH TREATMENT AND MANAGEMENT** | **CODE** |
| --- | --- |
| Very dissatisfied | 1 |
| Somewhat dissatisfied | 2 |
| Somewhat satisfied | 3 |
| Very satisfied | 4 |
| *Don’t know / Can’t say* | *99* |

1. Please read the following statements related to Myelofibrosis and indicate whether you agree strongly, agree somewhat, disagree somewhat or disagree strongly by selecting one option? **PLEASE ASSIGN A RATING BETWEEN 1-4 FOR EVERY STATEMENT**

| **STATEMENT** | **STRONGLY DISAGREE** | **SOMWHAT DISAGREE** | **SOMEWHAT AGREE** | **STRONGLY AGREE** |
| --- | --- | --- | --- | --- |
| Myelofibrosis is a blood cancer | 1 | 2 | 3 | 4 |
| Myelofibrosis may progress to a more serious condition | 1 | 2 | 3 | 4 |
| Myelofibrosis symptoms reduce a patient’s quality of life | 1 | 2 | 3 | 4 |
| The reimbursement of drug treatments plays an important role in the treatment I offer to my Myelofibrosis patients and/or is accepted by my patients | 1 | 2 | 3 | 4 |

1. How much do you feel that your Myelofibrosis patients **understand** the treatment goals? **SELECT ANY ONE OPTION**

| **UNDERSTANDING OF TREATMENT GOALS** | **CODE** |
| --- | --- |
| Not at all | 1 |
| Only a little | 2 |
| Somewhat | 3 |
| Completely | 4 |
| *Don’t know / Can’t say* | *99* |

1. How much do you feel that you and your Myelofibrosis patients **agree on** the treatment goals? **SELECT ANY ONE OPTION**

| **AGREEMENT ON TREATMENT GOALS** | **CODE** |
| --- | --- |
| Not at all | 1 |
| Only a little | 2 |
| Somewhat | 3 |
| Completely | 4 |
| *Don’t know / Can’t say* | *99* |

1. A. On an average, how much time do you spend counselling your patients / answering their queries at the time of diagnosis of their Myelofibrosis? **SELECT ANY ONE OPTION**

B. On an average, how much do you spend counselling your patients / answering their queries during their follow up consultations? **SELECT ANY ONE OPTION**

| **TIME SPENT COUNSELLING / ANSWERING PATIENT QUERIES** | **CODE- Q32A** | **CODE- Q32B** |
| --- | --- | --- |
| 5 minutes | 1 | 1 |
| 10 minutes | 2 | 2 |
| 15 minutes | 3 | 3 |
| 20 minutes | 4 | 4 |
| 30 minutes or more | 5 | 5 |

1. How satisfied are you with your communications with Myelofibrosis patients about their disease and treatment? **SELECT ANY ONE OPTION**

| **SATISFACTION WITH COMMUNICATION ON DISEASE AND TREATMENT** | **CODE** |
| --- | --- |
| Very dissatisfied | 1 |
| Somewhat dissatisfied | 2 |
| Somewhat satisfied | 3 |
| Very satisfied | 4 |

1. Based on your experience with your Myelofibrosis patients, which of the below scenarios / actions have you experienced / taken. **SELECT ALL THAT APPLY**

| **STATEMENT** | **CODE** |
| --- | --- |
| I ask my Myelofibrosis patients about their symptoms at every appointment | 1 |
| I create treatment plans or establish goals of therapy for my Myelofibrosis patients | 2 |
| I involve my Myelofibrosis patients in decisions about their treatments | 3 |
| I keep my Myelofibrosis patients informed about new treatment options | 4 |
| I provide my Myelofibrosis patients with disease brochures | 5 |
| I understand how much Myelofibrosis impacts my patient’s life | 6 |
| My Myelofibrosis patients don’t feel comfortable discussing their symptoms with me | 7 |
| My Myelofibrosis patients feel that I am genuinely concerned about helping them | 8 |
| My Myelofibrosis patients feel that I can really listen to their concerns and address their concerns | 9 |
| My patients always discuss their symptoms with me at every appointment | 10 |
| Myelofibrosis patients feel they are burdening me by discussing their symptoms | 11 |
| Myelofibrosis patients may be unsure of how to best describe their symptoms to their doctor | 12 |
| Myelofibrosis patients only discuss symptoms when their health condition is really bad | 13 |
| Myelofibrosis patients only discuss their symptoms with the nurse | 14 |
| Reviewing the patient’s blood count takes priority over a discussion on symptoms | 15 |
| There is not enough time during the appointment to discuss all the symptoms a patient is experiencing | 16 |

1. What do you consider to be the most important unmet need in your patient’s treatment? **SELECT ANY ONE OPTION**

| **UNMET NEEDS IN PATIENT TREATMENT** | **CODE** |  |
| --- | --- | --- |
| Cure | 1 |  |
| Effective drugs/therapy | 2 |  |
| Less expensive | 3 |  |
| Limit progression of disease | 4 |  |
| Prevent other disease | 5 |  |
| Reduce transfusions | 6 |  |
| Symptom improvement | 7 |  |
| *Others (please specify)* | *99* |  |
| **SECTION V: COVID-19 IMPACT AND PERCEPTIONS** | | |

1. Have you been redeployed to work in another clinical role because of the global COVID-19 pandemic? **SELECT ANY ONE OPTION**

| **REDPLOYMENT TO ANOTHER CLINICAL ROLE** | **CODE** |
| --- | --- |
| Yes - I have been redeployed to another full-time clinical role | 1 |
| Yes - I have been redeployed to cover another clinical role part time | 2 |
| No | 3 |

1. Was your redeployment temporary, or is it ongoing? **SELECT ANY ONE OPTION**

| **CURRENT STATUS OF REDPLOYMENT** | **CODE** |
| --- | --- |
| Temporary - I am now back in my usual role full time | 1 |
| Ongoing - the redeployment remains in effect | 2 |

1. On a scale of 1-5, 1 being the LEAST and 5 being the MOST, please provide a rating for the below statements about the COVID-19 pandemic, depending on the impact they have had on your current consultations with Myelofibrosis patients **PLEASE ASSIGN A RATING BETWEEN 1-5 FOR STATEMENT**

| **A lot less often than before the pandemic** | **A little less often than before the pandemic** | **No change** | **A little more often than before the pandemic** | **A lot more often than before the pandemic** |
| --- | --- | --- | --- | --- |
| 1 | 2 | 3 | 4 | 5 |

| **STATEMENTS ABOUT COVID-19** | **A lot less often than before the pandemic** | **A little less often than before the pandemic** | **No change** | **A little more often than before the pandemic** | **A lot more often than before the pandemic** |
| --- | --- | --- | --- | --- | --- |
| Impact of COVID-19 on discussion of QoL with my MF patients | 1 | 2 | 3 | 4 | 5 |
| Impact of COVID-19 on the frequency of my face-to-face interactions with my patients | 1 | 2 | 3 | 4 | 5 |
| Impact of COVID-19 on the frequency of my virtual / online interactions with my patients (e.g. Online consultations, WhatsApp calls, video calls) | 1 | 2 | 3 | 4 | 5 |

1. Overall, to what extent has the COVID-19 pandemic affected the overall quality of life of your myelofibrosis patients on a scale of 1-5 where 1 means “NOT AT ALL” and 5 means “A GREAT DEAL”? **PLEASE ASSIGN A RATING BETWEEN 1-5 FOR THE OVERALL IMPACT OF COVID-19 ON PATIENT QoL**

| **Much worse** | **A little worse** | **No different**  **(no impact)** | **A little better** | **Much better** |
| --- | --- | --- | --- | --- |
| 1 | 2 | 3 | 4 | 5 |

| **IMPACT OF COVID-19 ON PATIENT QoL** | **Much worse** | **A little worse** | **No different**  **(no impact)** | **A little better** | **Much better** |
| --- | --- | --- | --- | --- | --- |
|  | 1 | 2 | 3 | 4 | 5 |

1. To what extent do you feel COVID-19 is currently impacting the frequency of Myelofibrosis patient visits to your clinic/practice specifically when it comes to assessment and monitoring in relation to their condition? (e.g. Blood tests, spleen measurements) **SELECT ANY ONE OPTION**

| **EXTENT OF IMPACT OF COVID-19 ON ASSESSMENT AND MONITORING OF MYELOFIBROSIS** | **CODE** |
| --- | --- |
| No impact currently | 1 |
| Small impact/slightly fewer tests/assessments/monitoring visits and likely only temporary | 2 |
| Small impact/slightly fewer tests/assessments/monitoring visits and likely will be ongoing | 3 |
| Large impact/much fewer tests/assessments/monitoring visits and likely only temporary | 4 |

1. To what extent do you feel COVID-19 is currently impacting the treatment of Myelofibrosis patients? **SELECT ANY ONE OPTION**

| **EXTENT OF IMPACT OF COVID-19 ON MF TREATMENT** | **CODE** |
| --- | --- |
| No impact currently | 1 |
| Small impact/minor changes to treatment plans and likely only temporary | 2 |
| Small impact/minor changes to treatment plans and likely will be ongoing | 3 |
| Large impact/significant changes to treatment plans and likely only temporary | 4 |
| Large impact/significant changes to treatment plans and likely will be ongoing | 5 |

1. How has COVID-19 impacted the treatment of Myelofibrosis patients? **SELECT ALL THAT APPLY**

| **IMPACTS OF COVID-19 ON MF TREATMENT** | **CODE** |
| --- | --- |
| Delays in starting patients on new treatments | 1 |
| I have changed the way patients are treated (e.g. change in dose, treatment switch), specifically due to COVID-19 limitations | 2 |
| Patients are unable to collect drugs from the pharmacy | 3 |
| Patients have decided to not come in for consultation | 4 |
| None of the above | 5 |

# Supplementary Table 2: The patient questionnaire

| **SECTION 1: RESPONDENT DETAILS** |
| --- |

1. A. How old are you? **IN YEARS**

*Programming instructions: Range for this response is 18-99 Years*

|  | **AGE OF THE PATIENT** |
| --- | --- |

B. Could you please indicate the **highest level of education** that you have completed? **CODE APPROPRIATELY. SINGLE CODING ONLY**

| **EDUCATION** | **CODE** | **ACTION** |
| --- | --- | --- |
| Less than SSC/HSC | 01 | **TERMINATE** |
| SSC/HSC | 02 | **CONTINUE** |
| Graduate | 03 |  |
| Post-graduate master’s degree | 04 |  |
| Post-graduate master’s degree & Higher | 05 |  |

1. Which of the following best describes your **working status** currently? **CODE APPROPRIATELY. SINGLE CODING ONLY**

| **WORKING STATUS** | **CODE** |
| --- | --- |
| Corporate executive/ Middle level manager | 01 |
| Self -employed- Own business/shop owner | 02 |
| Freelancer | 03 |
| Homemaker | 04 |
| Retired | 05 |

1. What is your **Family status**? **CODE APPROPRIATELY. SINGLE CODING ONLY**

| **FAMILY TYPE** | **CODE** |
| --- | --- |
| Conjugal unit (2 individuals) | 01 |
| Nuclear (small family with children/parents) | 02 |
| Joint or extended family (3 lateral/vertical generations) | 03 |

1. What is your **household size**?

|  |  |  | **RECORD NO. OF FAMILY MEMBERS** |
| --- | --- | --- | --- |

1. Please indicate the **total annual household income**. **SINGLE CODE ONLY**

| **HOUSEHOLD INCOME** | **CODE** |
| --- | --- |
| < 5 lakhs | 01 |
| 5- 10 lakhs | 02 |
| > 10 lakhs | 03 |

1. Could you please indicate the **level of education of the Chief Wage Earner (CWE)** of your household? By Chief Wage Earner, we mean the person who contributes maximum to the expenditure of your household? **SINGLE CODE ONLY**

| **EDUCATION** | **CODE** |
| --- | --- |
| Uneducated | 01 |
| School up to 4 years | 02 |
| School 5 to 9 years | 03 |
| SSC/HSC | 04 |
| Some College, but not graduate | 05 |
| Graduate/ Post-graduate – General | 06 |
| Graduate/Post-Graduate- Professional | 07 |

1. From the below list of items, please indicate which of these **items do you own**? (It could be owned by you or any of your family members)? **MULTIPLE CODING POSSIBLE**

| **ITEMS** | **CODE** |
| --- | --- |
| Electricity connection | 01 |
| Ceiling Fan | 02 |
| LPG Stove | 03 |
| Two-wheeler vehicle | 04 |
| Colour TV | 05 |
| Refrigerator | 06 |
| Washing Machine | 07 |
| Personal Computer/Laptop | 08 |
| Car/Jeep/Van | 09 |
| Air conditioner | 10 |
| Agricultural land (currently under cultivation or plantation) | 11 |

| **EDUCATION 🡪**  **NUMBER OF DURABLES** 🡻 | **Uneducated** | **School up to 4 years** | **School 5-9 years** | **SSC/ HSC** | **Some college but not graduate** | **Graduate/ Post-**  **Graduate:**  **General** | **Graduate/**  **Post-**  **Graduate:**  **Professional** |
| --- | --- | --- | --- | --- | --- | --- | --- |
|  | (1) | (2) | (3) | (4) | (5) | (6) | (7) |
| None | E3 | E2 | E2 | E2 | E2 | E1 | D2 |
| 1 | E2 | E1 | E1 | E1 | D2 | D2 | D2 |
| 2 | E1 | E1 | D2 | D2 | D1 | D1 | D1 |
| 3 | D2 | D2 | D1 | D1 | C2 | C2 | C2 |
| 4 | D1 | C2 | C2 | C1 | C1 | B2 | B2 |
| 5 | C2 | C1 | C1 | B2 | B1 | B1 | B1 |
| 6 | C1 | B2 | B2 | B1 | A3 | A3 | A3 |
| 7 | C1 | B1 | B1 | A3 | A3 | A2 | A2 |
| 8 | B1 | A3 | A3 | A3 | A2 | A2 | A2 |
| 9+ | B1 | A3 | A3 | A2 | A2 | A1 | A1 |

1. At what age were you diagnosed with Myelofibrosis? **IN YEARS**

*Programming instructions: Range for this response is 0-90 Years*

|  | **AGE WHEN DIAGNOSED WITH MYELOFIBROSIS** |
| --- | --- |

1. What is your gender? **PLEASE SELECT THE GENDER OF THE PATIENT**

| **GENDER** | **CODE** |
| --- | --- |
| Male | 1 |
| Female | 2 |
| Other | 99 |

1. Please rate the below symptoms based on the level of difficulty you have faced in the past week because of them **PLEASE ASSIGN A RATING BETWEEN 1-10 FOR EACH OF THE BELOW SYMPTOMS**

| **MPN-10 QUESTIONNAIRE** | **No Fatigue** |  |  |  |  |  |  |  |  | **Worst Imaginable** |
| --- | --- | --- | --- | --- | --- | --- | --- | --- | --- | --- |
| Fatigue (Tiredness / Weariness) | 1 | 2 | 3 | 4 | 5 | 6 | 7 | 8 | 9 | 10 |
|  | **Absent** |  |  |  |  |  |  |  |  | **Daily** |
| Fever (>100◦F) | 1 | 2 | 3 | 4 | 5 | 6 | 7 | 8 | 9 | 10 |
|  | **Absent** |  |  |  |  |  |  |  |  | **Worst Imaginable** |
| Filling up quickly when you eat (early satiety) | 1 | 2 | 3 | 4 | 5 | 6 | 7 | 8 | 9 | 10 |
| Abdominal discomfort | 1 | 2 | 3 | 4 | 5 | 6 | 7 | 8 | 9 | 10 |
| Inactivity | 1 | 2 | 3 | 4 | 5 | 6 | 7 | 8 | 9 | 10 |
| Problems with concentration | 1 | 2 | 3 | 4 | 5 | 6 | 7 | 8 | 9 | 10 |
| Night sweats | 1 | 2 | 3 | 4 | 5 | 6 | 7 | 8 | 9 | 10 |
| Itching (pruritus) | 1 | 2 | 3 | 4 | 5 | 6 | 7 | 8 | 9 | 10 |
| Bone Pain (diffuse not joint pain or arthritis) | 1 | 2 | 3 | 4 | 5 | 6 | 7 | 8 | 9 | 10 |
| Unintentional weight loss in the last 6 months | 1 | 2 | 3 | 4 | 5 | 6 | 7 | 8 | 9 | 10 |

1. How would you describe your current state of health? **SELECT ANY ONE OPTION**

| **CURRENT STATE OF HEALTH** | **CODE** |
| --- | --- |
| Excellent | 1 |
| Very good | 2 |
| Good | 3 |
| Fair | 4 |
| Poor | 5 |
| Very poor | 6 |

1. A. Rank the following statements on a scale of 1-10, where 1 means NOT AT ALL and 10 means A GREAT DEAL as they have occurred during the past month, as a result of your Myelofibrosis**: GIVE A RATING BETWEEN 1-10 FOR EACH STATEMENT**

| **NOT AT ALL** |  |  |  |  |  |  |  |  | **A GREAT DEAL** |
| --- | --- | --- | --- | --- | --- | --- | --- | --- | --- |
| 1 | 2 | 3 | 4 | 5 | 6 | 7 | 8 | 9 | 10 |

| **STATEMENTS WITH RESPECT TO MYELOFIBROSIS** | **NOT AT ALL** |  |  |  |  |  |  |  |  | **A GREAT DEAL** |
| --- | --- | --- | --- | --- | --- | --- | --- | --- | --- | --- |
| I feel embarrassed of my physical appearance due to my disease | 1 | 2 | 3 | 4 | 5 | 6 | 7 | 8 | 9 | 10 |
| I have been irritable or unusually angry | 1 | 2 | 3 | 4 | 5 | 6 | 7 | 8 | 9 | 10 |
| I have felt depressed | 1 | 2 | 3 | 4 | 5 | 6 | 7 | 8 | 9 | 10 |
| I have felt discouraged | 1 | 2 | 3 | 4 | 5 | 6 | 7 | 8 | 9 | 10 |
| I have had trouble coping with the stress I have been having | 1 | 2 | 3 | 4 | 5 | 6 | 7 | 8 | 9 | 10 |
| I have noticed a change in my appetite | 1 | 2 | 3 | 4 | 5 | 6 | 7 | 8 | 9 | 10 |
| My condition has caused emotional hardship for me | 1 | 2 | 3 | 4 | 5 | 6 | 7 | 8 | 9 | 10 |
| My condition has caused financial hardship for me | 1 | 2 | 3 | 4 | 5 | 6 | 7 | 8 | 9 | 10 |
| My condition has caused physical hardship for me | 1 | 2 | 3 | 4 | 5 | 6 | 7 | 8 | 9 | 10 |
| My condition is controlling my life | 1 | 2 | 3 | 4 | 5 | 6 | 7 | 8 | 9 | 10 |
| My sleeping habits have negatively changed | 1 | 2 | 3 | 4 | 5 | 6 | 7 | 8 | 9 | 10 |

1. Thinking of how your Myelofibrosis affects you emotionally please rank the following statements on a scale of 1-10, where 1 means NOT AT ALL and 10 means A GREAT DEAL. **GIVE A RATING BETWEEN 1-10 FOR EACH STATEMENT**

| **NOT AT ALL** |  |  |  |  |  |  |  |  | **A GREAT DEAL** |
| --- | --- | --- | --- | --- | --- | --- | --- | --- | --- |
| 1 | 2 | 3 | 4 | 5 | 6 | 7 | 8 | 9 | 10 |

| **EMOTIONAL STATUS DUE TO MYELOFIBROSIS** | **NOT AT ALL** |  |  |  |  |  |  |  |  | **A GREAT DEAL** |
| --- | --- | --- | --- | --- | --- | --- | --- | --- | --- | --- |
| I am frustrated tolerating the symptoms of my disease | 1 | 2 | 3 | 4 | 5 | 6 | 7 | 8 | 9 | 10 |
| I am losing hope in the fight against my illness | 1 | 2 | 3 | 4 | 5 | 6 | 7 | 8 | 9 | 10 |
| I am unsatisfied with how I am coping with my illness | 1 | 2 | 3 | 4 | 5 | 6 | 7 | 8 | 9 | 10 |
| I am unsure if I am being assessed and treated properly | 1 | 2 | 3 | 4 | 5 | 6 | 7 | 8 | 9 | 10 |
| I feel frustrated having a rare, long term illness | 1 | 2 | 3 | 4 | 5 | 6 | 7 | 8 | 9 | 10 |
| I feel helpless because of my disease | 1 | 2 | 3 | 4 | 5 | 6 | 7 | 8 | 9 | 10 |
| I feel nervous | 1 | 2 | 3 | 4 | 5 | 6 | 7 | 8 | 9 | 10 |
| I feel sad | 1 | 2 | 3 | 4 | 5 | 6 | 7 | 8 | 9 | 10 |
| I often feel worse than my physician is aware of | 1 | 2 | 3 | 4 | 5 | 6 | 7 | 8 | 9 | 10 |
| I worry about dying / worry that my condition will get worse | 1 | 2 | 3 | 4 | 5 | 6 | 7 | 8 | 9 | 10 |

1. To what extent, on a scale of 1-10, where 1 means NOT AT ALL and 10 means A GREAT DEAL does your Myelofibrosis interfere with the following activities in your life: **GIVE A RATING BETWEEN 1-10 FOR EACH ACTIVITY**

| **NOT AT ALL** |  |  |  |  |  |  |  |  | **A GREAT DEAL** |
| --- | --- | --- | --- | --- | --- | --- | --- | --- | --- |
| 1 | 2 | 3 | 4 | 5 | 6 | 7 | 8 | 9 | 10 |

| **EFFECT OF MYELOFIBROSIS ON DAILY ACTIVITITES** | **NOT AT ALL** |  |  |  |  |  |  |  |  | **A GREAT DEAL** |
| --- | --- | --- | --- | --- | --- | --- | --- | --- | --- | --- |
| My condition has interfered with my daily activities | 1 | 2 | 3 | 4 | 5 | 6 | 7 | 8 | 9 | 10 |
| My condition has interfered with my family or social life | 1 | 2 | 3 | 4 | 5 | 6 | 7 | 8 | 9 | 10 |
| My condition has interfered with my sex life | 1 | 2 | 3 | 4 | 5 | 6 | 7 | 8 | 9 | 10 |
| Pain and discomfort have caused me to limit my activities | 1 | 2 | 3 | 4 | 5 | 6 | 7 | 8 | 9 | 10 |
| My condition has interfered with my relationship with my caregiver | 1 | 2 | 3 | 4 | 5 | 6 | 7 | 8 | 9 | 10 |

| **SECTION 2: IMPACT OF DISEASE ON DAILY LIVING** |
| --- |

1. In the last 30 days, how many times did you have to cancel planned or scheduled activities (i.e., lunch with friends, shopping trip, appointment, etc.) because of your Myelofibrosis? **PLEASE ENTER A NUMBER**

*Programming Instructions: Range for this response is 0-100*

|  | **NUMBER OF TIMES PLANNED / SCHEDULED ACTIVITIES HAVE BEEN CANCELLED IN THE LAST 30 DAYS** |
| --- | --- |

1. In the last 30 days, how many times did you stay in bed all or most of the day because of your Myelofibrosis? **PLEASE ENTER A NUMBER**

*Programming Instructions: Range for this response is 0-30*

|  | **NUMBER OF TIMES STAYED IN BED ALL OR MOST OF THE DAY** |
| --- | --- |

1. As a result of your Myelofibrosis, have you ever faced any of the below situations? **SELECT ANY ONE OPTION FOR EACH SITUATION**

**IF YOU HAVE SELECTED YES FOR** <Reduced hours at work>, **PLEASE ENTER THE NUMBER OF HOURS/WEEK THAT WERE REDUCED BECAUSE OF YOUR MYELOFIBROSIS**

| **SITUATION** | **YES** | **NO** | **NA** | **By how many hours per week** |
| --- | --- | --- | --- | --- |
| Reduced your hours at work | 1 | 2 | 3 | __ hours |
| Voluntarily terminated your job | 1 | 2 | 3 |  |
| Been involuntarily terminated from your job | 1 | 2 | 3 |  |
| Gone on disability living allowance | 1 | 2 | 3 |  |
| Taken early retirement | 1 | 2 | 3 |  |
| Taken a lower paid job | 1 | 2 | 3 |  |

1. How often do you rely on someone (i.e. caregiver) to assist you with your activities of daily living due to your Myelofibrosis? **SELECT ANY ONE OPTION**

*Assistance with activities of daily living can range from a few hours of shopping and cleaning to intensive medical or personal care. Tasks can include shopping, house cleaning, cooking, giving medications, toileting assistance and so forth. The person who assists you with your activities of daily living will be referred to as “****caregiver****” in the following survey.*

| **ASSISTANCE WITH ACTIVITIES OF DAILY LIVING** | **CODE** |
| --- | --- |
| Never | 1 |
| Rarely | 2 |
| Sometimes | 3 |
| Often | 4 |

***ASK Q12-Q16 TO ONLY THOSE RESPONDENTS WHO HAVE CODED 2,3 OR 4 IN Q11, ELSE SKIP TO Q17***

1. Who is the main caregiver who helps you with your Myelofibrosis? **SELECT ANY ONE OPTION**

| **PRIMARY CAREGIVER** | **CODE** |
| --- | --- |
| Spouse/partner | 1 |
| Son/daughter | 2 |
| Parent | 3 |
| Sibling | 4 |
| Other relative | 5 |
| Friend/neighbor | 6 |
| Paid nurse or home healthcare professional | 7 |
| *Others (please specify)* | *99* |

1. What kind of help do you require from your main caregiver due to your Myelofibrosis? **SELECT ALL THAT APPLY**

| **NATURE OF ASSISTANCE WITH ACTIVITIES** | **CODE** |
| --- | --- |
| Companionship (e.g., talking, reading, keeping company) or supervision, emotional support or encouragement | 1 |
| Transportation (e.g., driving to doctor’s appointments, driving for errands) | 2 |
| Homemaking (e.g., shopping, cleaning, preparing meals) | 3 |
| Personal care assistance (e.g., feeding, bathing, toileting, dressing, grooming) | 4 |
| Healthcare assistance (e.g., help with medications, wound care, researching condition) | 5 |
| Managing finances (e.g., paying bills, managing budget) | 6 |
| Help plan and organize everyday activities | 7 |
| *Others (please specify)* | *99* |

1. In the past 7 days, how many hours have you received help from your main caregiver to help you with activities associated with your Myelofibrosis? **PLEASE ENTER A NUMBER**

*Programming Instructions: Range for this response is 0-168 hours*

|  | **NUMBER OF HOURS RECEIVING HELP FROM PRIMARY CAREGIVER** |
| --- | --- |

***ASK ONLY TO THOSE RESPONDENTS WHO HAVE CODED 1-6 IN Q12, ELSE SKIP TO Q17***

1. Is your caregiver currently employed? **SELECT ANY ONE OPTION**

| **YES** | **NO** |
| --- | --- |

***ASK ONLY TO THOSE RESPONDENTS WHO HAVE CODED 1-6 IN Q12, ELSE SKIP TO Q17***

1. As a result of your Myelofibrosis has your caregiver ever faced any of the below situations with regards to their employment. **SELECT YES/NO FOR EACH SITUATION**

| **SITUATION** | **YES** | **NO** |
| --- | --- | --- |
| Changed from full-time to part-time employment | 1 | 2 |
| Reduced his/her hours at work | 1 | 2 |
| Taken early retirement | 1 | 2 |
| Voluntarily terminated his/her job | 1 | 2 |
| Been involuntarily terminated from their job | 1 | 2 |
| Considered terminating his/her job (but did not terminate for reasons such as health insurance coverage, etc.) | 1 | 2 |

***ASK ALL RESPONDENTS***

1. On a scale of 1 to 10, where 1 means NO IMPACT and 10 means MAJOR IMPACT (1 = No Impact, 10 = Major Impact), what impact, if any, has your Myelofibrosis had on your caregiver? **GIVE A RATING BETWEEN 1-10**

| **IMPACT OF MYELOFIBROSIS ON PRIMARY CAREGIVER** | **No Impact** |  |  |  |  |  |  |  |  | **Major Impact** |
| --- | --- | --- | --- | --- | --- | --- | --- | --- | --- | --- |
|  | 1 | 2 | 3 | 4 | 5 | 6 | 7 | 8 | 9 | 10 |

**Supplementary Figure S1**: Referral Dynamics of Patients Diagnosed with Primary MF, Post-PV And Post-ET


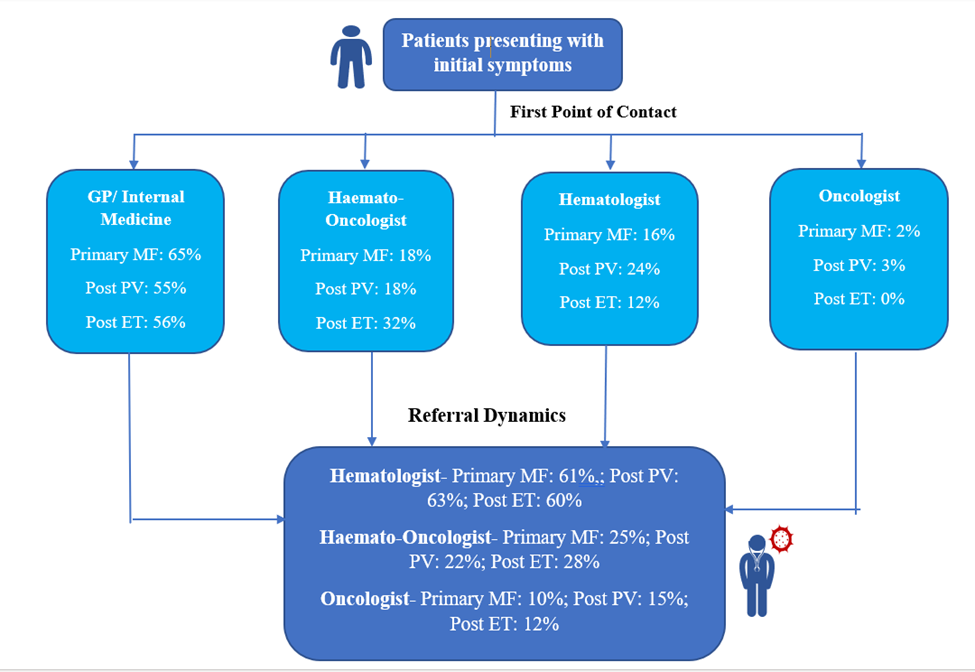


ET- Essential thrombocythemia; GP- General Physician; MF- Myelofibrosis; PV- Polycythemia vera

**Supplementary Figure S2:** Adapted from “A Double-Blind, Placebo-Controlled Trial of Ruxolitinib for Myelofibrosis” by Verstovsek S, N Engl J Med 2012;366:799-807.


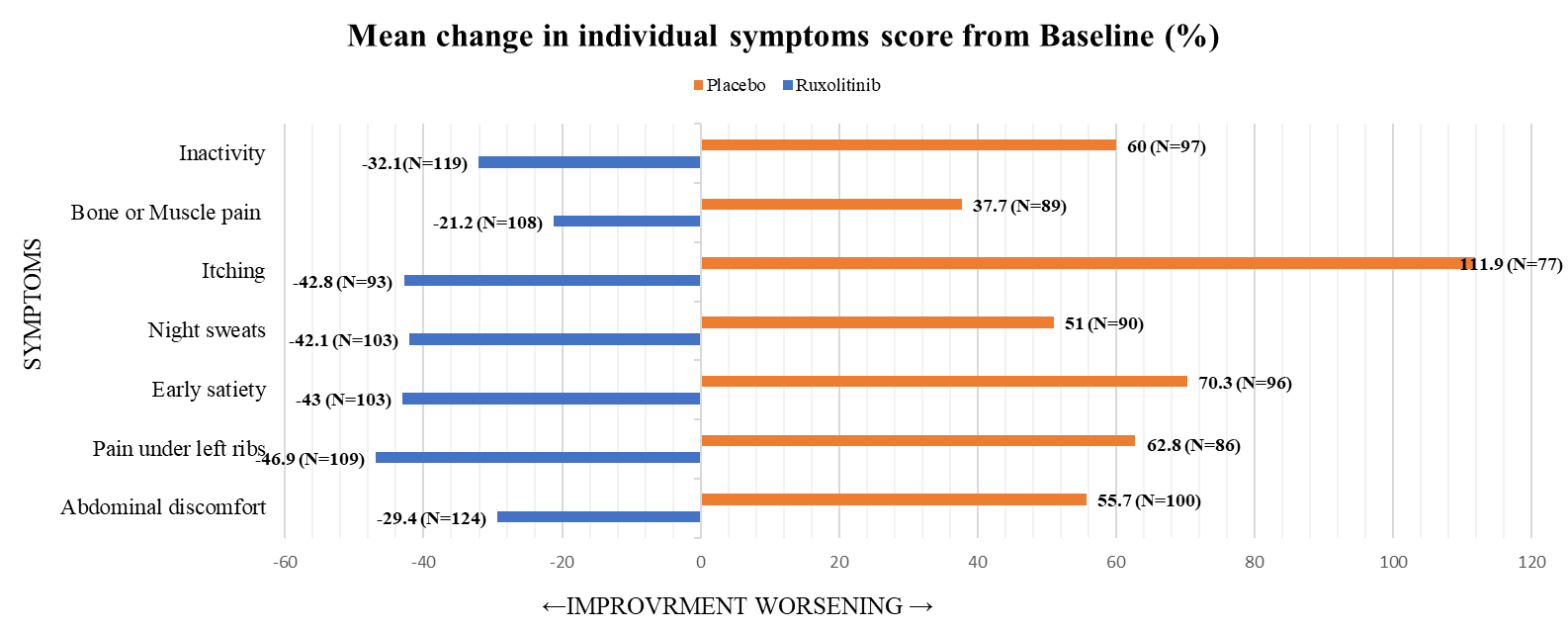


**Supplementary Figure S3:** Adapted from “JAK inhibition with Ruxolitinib versus best available therapy for myelofibrosis.” by Harrison C, N Engl J Med 2012; 366(9):787-98


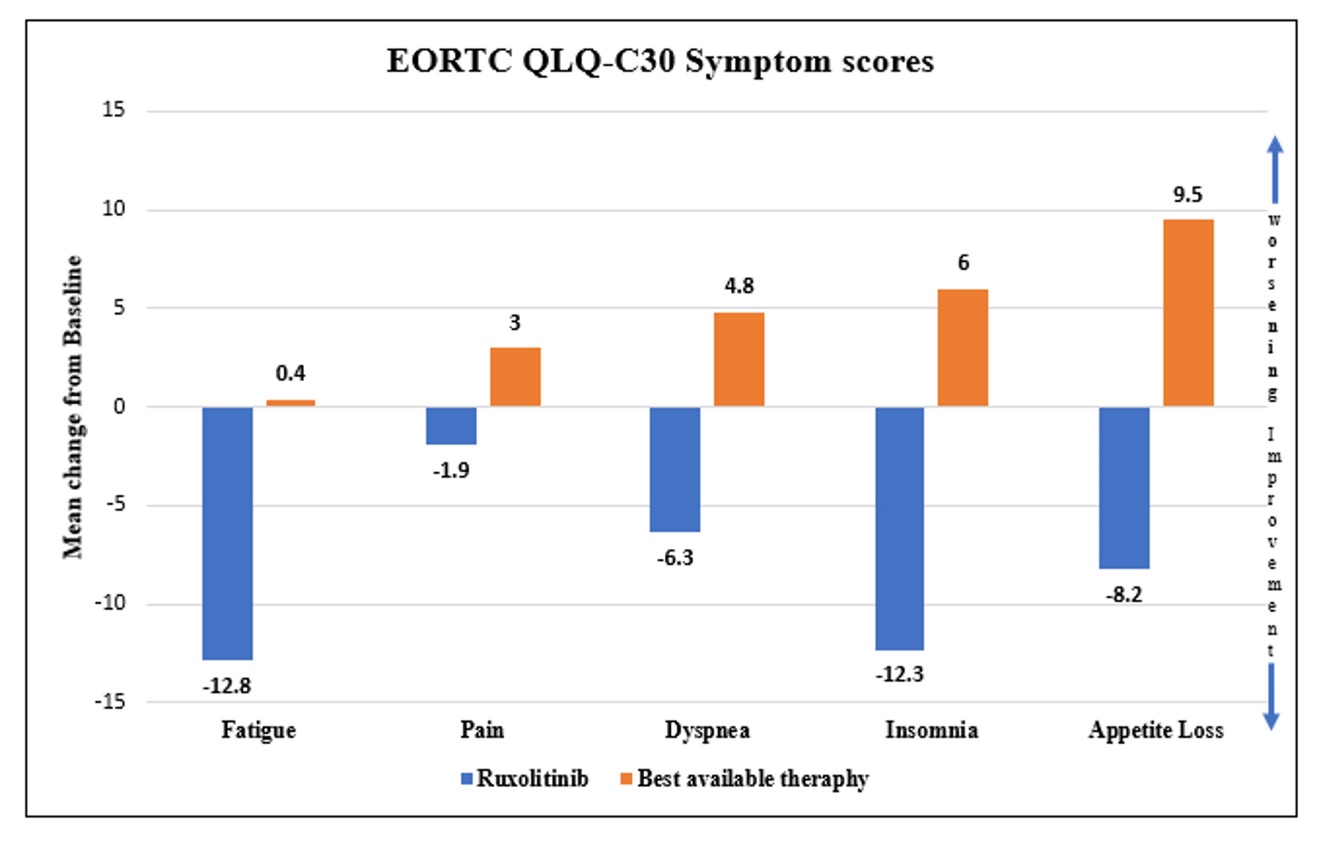

Supplement: Supplementary file 1 — Supplementary Material 1 [file 12885_2025_14476_MOESM1_ESM.docx]
